# Inkjet-Printed Aqueous Graphene Suspensions on Flexible Substrates for Multimodal Wearable Continuous Health Monitoring

**DOI:** 10.1021/acsnanoscienceau.5c00195

**Published:** 2026-04-22

**Authors:** Seyed Sajjad Mirbakht, Faruk Ballipinar, Burcu Arman Kuzubasoglu, Melih Can Tasdelen, Saygun Guler, Murat Kaya Yapici

**Affiliations:** † Faculty of Engineering and Natural Sciences, Sabanci University, 34956 Istanbul, Türkiye; ‡ Sabanci University Micro/Nano Devices and Systems Lab (SU-MEMS), 52991 Sabanci University, 34956 Istanbul, Türkiye; § Sabanci University SUNUM Nanotechnology Research Center, 34956 Istanbul, Türkiye; ∥ Department of Electrical and Computer Engineering, University of Washington, Seattle, Washington 98195, United States

**Keywords:** biopotential monitoring, functional inks, printed electronics, neural signals, printing process optimization

## Abstract

Flexible and gel-free dry electrophysiological electrodes offer excellent alternatives to the benchmark with better skin conformality and dispensing with the hydrogel electrolyte layer, where the integrity of recorded signals highly depends on the resilience of this layer. However, the implicit complex fabrication methods and resource-intensive nature of existing dry electrodes, designed for single use, necessitate additional technological innovations. This paper presents an original integration of inkjet-printing, graphene, and poly­(ethylene terephthalate) (PET) substrates to create flexible, inexpensive, and reusable dry electrodes for monitoring human physiological biopotential signals, including ECG, EEG, EOG, and EMG. The electrodes exhibited high electrical conductivity (80 Ω/□) and maintained their performance even after 100 cyclic bends. Additionally, they demonstrated robust signal collection even 90 days postfabrication, with reusability and strong graphene-substrate bonding verified through Scotch tape experiments. Assessing against gel-based commercial electrodes, a 99.34% correlation ratio was obtained from the recorded ECG signals. Similarly, a correlation ratio of 95.14 ± 4.75% was achieved with 10 diverse participants. The capability of these electrodes to effectively record neural signals in practical applications was demonstrated in real-time detection of sleep and drowsiness in car drivers. These findings highlight the potential of inkjet-printed graphene electrodes in advancing wearable health monitoring technologies.

## Graphical abstract

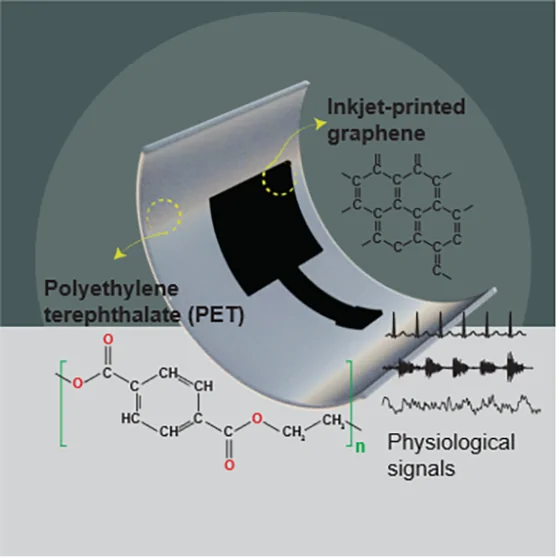

## 1 Introduction

In the past decade, flexible electronic devices have arisen for various applications including energy storage and harvesting (e.g., supercapacitors, triboelectric nanogenerators-TENGs),^1,2^ lighting and display applications (e.g., OLEDs),^3^ health monitoring, diagnosis and preventive care (e.g., skin patches, tattoo electronics).^4–6^ Thanks to their miniature form-factor, suitability for pick-and-place integration with surface mount low-power CMOS,^7^ massive scalability and adaptability for roll-to-roll (R2R) manufacturing,^8^ flexible hybrid electronics (FHEs) has emerged as a disruptive technology.^9,10^ Especially, in the personalized wellness domain, where continuous monitoring and acquisition of data from daily activities is needed, low-power wearable systems that can conform to the body even in dynamic conditions are critical. To that end, various substrate—functional layer combinations have been investigated to realize flexible health monitoring devices by utilizing existing fabrication techniques typically with certain modifications and integration strategies.^11,12^ However, a significant manufacturing bottleneck remains: balancing the high throughput of these systems with economic cost-effectiveness and environmental sustainability, particularly concerning material waste and the use of nonrecyclable substrates.

Metals such as silver (Ag) and copper (Cu), oftentimes coming in paste or ink formulation with suitable viscosity, are the most widely used functional materials in flexible electronics due to their high conductivity.^13,14^ While these materials have been used as standard, 2D layered materials, especially graphene and its derivatives like graphene oxide (GO) and reduced GO (rGO), offer the unique combination of biocompatibility and environmental sustainability needed for “single-use” or “disposable” skin-interfacing sensors. The appeal of such materials derives both from the sustainability perspective due to their cost-effective solution-based preparation methods,^15^ scalability,^16^ environmental friendliness and suitability for the circular economy;^17^ and also from the functionality perspective owing to physiochemical properties including mechanical flexibility and stretchability,^18^ large surface area for functionalization and drug loading,^19^ biocompatibility,^20^ and abundant oxygen-containing groups for electrochemical sensing,^21^ to name a few.

Despite these advantages, the transition from graphene “source material” to a functional device is governed by the compatibility between the material’s formulation and the chosen fabrication process. While techniques such as screen printing,^22^ aerosol jet,^23^ and gravure printing^24^ have been utilized, they often face challenges regarding material waste, high setup costs, or the need for physical masks. Inkjet printing distinguishes itself from the above-mentioned methods as a fully digital, additive, and mask-less technique, offering high-resolution patterning with nearly zero material waste—a critical factor for scalable, sustainable manufacturing.

Building on these advantages, inkjet-printed graphene can be effectively utilized in monitoring various electrophysiological signals through flexible dry electrode forms. Key examples of such biosignals are electrocardiography (ECG),^25,26^ electromyography (EMG),^27,28^ electroencephalography (EEG),^29,30^ and electrooculography (EOG),^31,32^ which provide excellent assessment tools in the diagnosis and treatment of cardiovascular diseases,^33^ rehibiliation,^34^ neurological disorders,^35^ and human-machine interface (HMI) applications,^36^ respectively. To enable high-quality acquisition of electrophysiological signals, electrodes interfacing with skin must possess specific properties. (1) Low skin-electrode impedance is crucial for collecting signals with a high signal-to-noise ratio (SNR).^37^ The importance of this property is evident in EEG signal recording, in which high skin-electrode impedance can result in complete signal loss at the skin and electrode interface before reaching the electronic circuitry.^38^ (2) High electrical conductivity is an interdependent property with electrode impedance that grants a reliable signal transfer from the skin to electronic devices.^39^ (3) Electrode adaptation to skin deformations—achieved through the use of flexible materials—reduces motion artifacts associated with natural body movements and skin deformations, ultimately enhancing comfort and wearability.^40,41^ (4) The biocompatibility of the materials that come into direct contact with the skin is pivotal for minimizing the risk of skin irritations and trauma.^42^

Despite significant research into the fabrication and performance evaluation of dry electrodes, past efforts have usually failed to address an ongoing paradigm in the development of biomedical devices which demands: (1) scalable fabrication techniques that can cater to a large population while keeping the final product costs low; (2) the use of recyclable materials to mitigate electronic waste; and (3) minimum dependence on natural resources. The current reliance on complex fabrication processes (e.g., cleanroom-based processes),^43,44^ nonrecoverable materials (e.g., poly­(dimethylsiloxane) (PDMS) and thermoplastic polyurethane (TPU)),^45,46^ and finite natural elements (e.g., copper, silver and gold) for products designed for a single-use^47,48^ presents challenges that require careful and innovative solutions. This is where additive, inkjet printing of 2D materials like graphene enter the scene, however with critical technical challenges in manufacturability and device integration which have been largely unmet thus far. Essentially, the success of inkjet printing approach depends fundamentally on the rheological properties of the graphene ink. Unlike other printing methods, inkjet printing requires a meticulous balance of viscosity and surface tension to ensure stable drop-on-demand ejection and eventually successful print formation.

To address this issue, herein we detail, to the best of our knowledge, the first systematic report on flexible inkjet-printed dry graphene electrodes on recyclable and affordable polyethylene terephthalate (PET) substrates, achieved by fine-tuning and optimizing the inkjet-printing process. We demonstrate the interplay between ink rheology, substrate surface properties, inkjet printing waveform and jetting profile, to report the optimized conditions for successful printing of dry, flexible graphene electrodes on PET, gathered as a culmination of extensive process development trials. To demonstrate the functionality of these flexible dry electrodes, we employ them in capturing a wide range of human electrophysiological signals, including ECG, EMG, EEG, and EOG, when interfaced with the skin. The performance of the printed graphene electrodes demonstrated a high correlation ratio with simultaneously recorded ECG signals from commercial Ag/AgCl electrodes, with a similar high correlation ratio observed between printed AgNP electrodes and Ag/AgCl electrodes. Additionally, the generalizability of the printed electrodes in recording electrophysiological signals in a larger population, composed of participants with varying ages, genders, and BMIs, showed statistical results similar to those of clinical-grade electrodes. The electrodes also exhibited high conductivity, excellent resilience to cyclic bending when evaluated by recording ECG, and consistent bonding between the printed materials and substrates, as assessed by scotch tape experiments. Finally, to demonstrate the applicability of the reported electrodes in real-life wearable applications, the printed graphene electrodes showed high accuracy in recording neural signals for detecting drowsiness in car drivers, thereby contributing to accident prevention.

## 2 Results and Discussion

### 2.1 Development of Inkjet-Printed Flexible Graphene Dry Electrodes

Figure 1a showcases the inkjet-printed dry electrodes on flexible and transparent PET substrates, highlighting their remarkable adaptability to various mechanical deformations such as twisting and bending. This flexibility underscores the potential of these electrodes in continuous daily health monitoring systems, where “user comfort” and “monitoring accuracy” are paramount. The highly conductive printed dry electrodes, with an active area of 2.5 cm × 2.5 cm, are capable of recording a wide range of human physiological biopotential signals, including ECG, EEG, EMG, and EOG (Figure 1b,c). Indeed, the effective performance of printed graphene electrodes in recording EEG signals can be utilized in daily wearable applications, such as detecting sleep and drowsiness in car drivers to prevent fatal accidents (Figure 1d). Achieving such high performance could only be possible through precise tuning of the ink rheological properties and optimization of the printing process to ensure a highly controllable and precise drop-on-demand inkjet profile (Movie S1 and Figure S1). To evaluate the performance and sustainability of our platform relative to the state-of-the-art, a comparative analysis was conducted against highly cited graphene-based biopotential electrodes (Table S1).^49–55^ While inkjet-printed graphene electrodes remain rare in current literature, our work distinguishes itself by enabling a wide range of signal acquisition—covering ECG, EMG, EEG, and EOG—on a highly recyclable PET substrate. Furthermore, we demonstrated the practical utility of these sensors by implementing a drowsiness detection system based on EEG signals, highlighting the clinical potential of the proposed inkjet-printed platform. Unlike single-use commercial alternatives, the reported electrodes exhibit excellent reusability, highlighting the clinical and environmental potential of this inkjet-printed platform.

**Figure 1:**
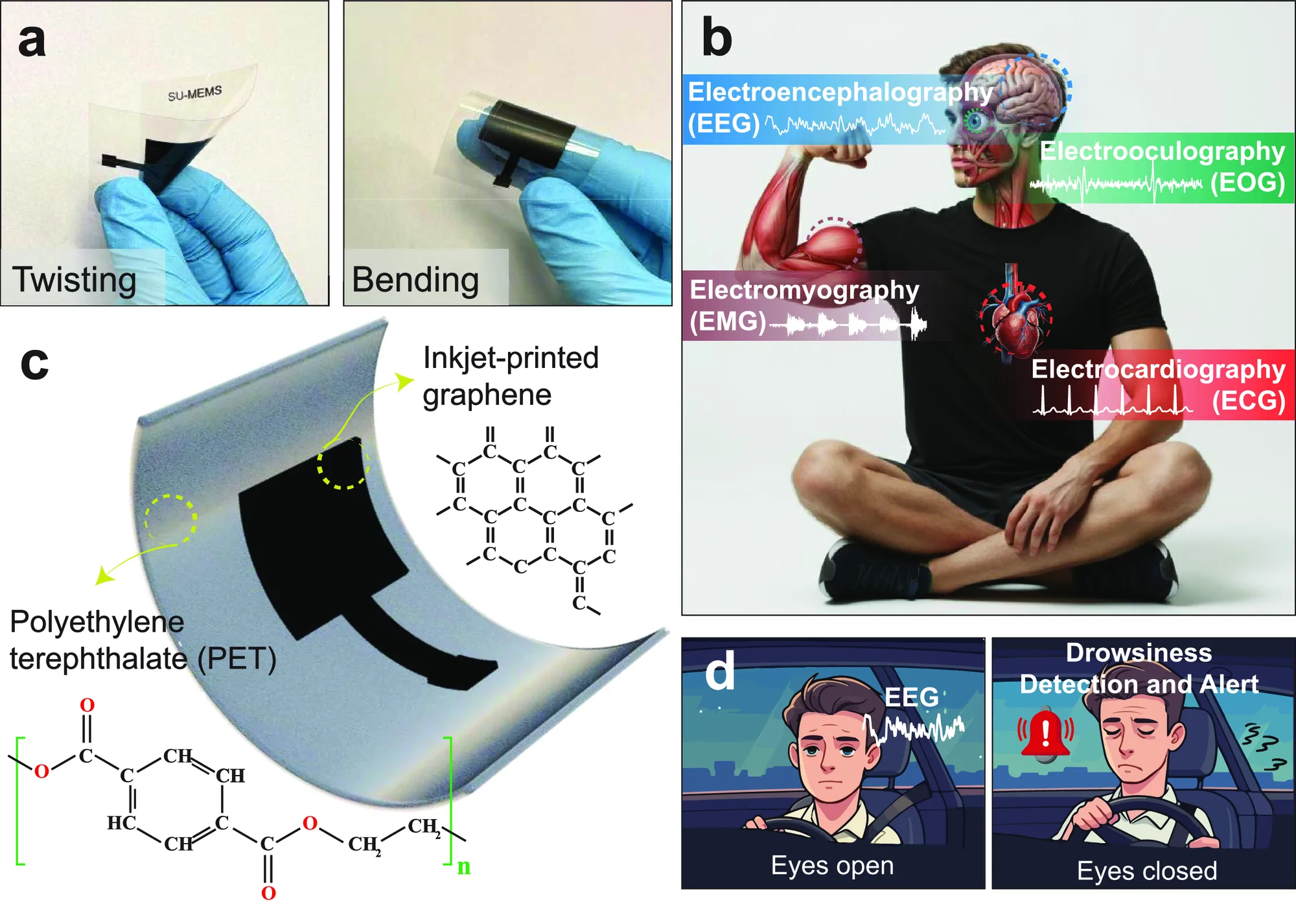
Schematic illustration of the inkjet-printed graphene dry electrodes on PET substrates for various physiological biopotential signal monitoring. (a) Mechanical deformations of printed graphene dry electrodes, including twisting and bending, showcasing their flexibility. (b) Potential applications of the printed dry electrodes in recording neural signals (EEG), oculography signals (EOG), neuromuscular signals (EMG), and cardiac signals (ECG). (c) Schematic demonstration of the printed dry electrodes, featuring a 2.5 cm square region for skin interfacing, followed by a filament for electrical connections. (d) Practical application of printed graphene electrodes in real-time detection of sleep and drowsiness in car drivers. [*DALL-E 3 tool was used for rendering the graphics in parts (b) and (d)]*.

### 2.2 Graphene Ink Characterization

To assess the stability of particle suspensions in conductive inks, the interaction energy between particles can be measured using Zeta (ζ) potential. This measurement evaluates the level of electrostatic repulsion between similarly charged neighboring particles.^56^ A large positive or negative Zeta potential indicates strong repulsion, which results in lower chances of aggregation and, consequently, higher stability of the ink. Zeta potential values are categorized as follows: 0–10 mV indicates high instability, 10–20 mV suggests relative stability, 20–30 mV is considered moderate stability, and values greater than 30 mV are classified as high stability.^57^ As illustrated in Figure S2, the zeta potential of the graphene ink is measured as −16.3 ± 3.56 mV, indicating that the graphene dispersion is relatively stable.

The jetting profile and the fluidic behaviors of the ink on the printing substrate (such as spreadability) are closely related to the rheological properties of the ink, which include viscosity and surface tension. These parameters uniquely and independently influence the quality of inkjet printing.^58^ For instance, high pulse pressure is needed for jetting when viscosity is high, while low viscosity can lead to dripping. Conversely, higher surface tension results in more stable drop formations and better wettability on the substrate, whereas lower surface tension can cause the formation of satellites.^59^ Therefore, optimizing these properties is crucial for achieving high-quality printing. The combined effects of surface tension and viscosity can be evaluated using the inverse of the Ohnesorge number, (*Z*), as represented in the equation below

1 $$Z={\mathrm{Oh}}^{-1}=\frac{\sqrt{\gamma \rho L}}{\eta }$$

where *Oh* is the Ohnesorge number, γ is the surface tension of the ink, ρ is the ink density, *L* represents the characteristic length related to the nozzle diameter, and η is the dynamic viscosity.^60^ A fluid is inherently capable of forming droplets and suitable for printing when its inverse Ohnesorge number ranges from 1 to 10.^61^ A low *Z* value, due to a high viscous force, necessitates a high pulse pressure for jetting, which can prevent the ejection of droplets. Conversely, a high *Z* value results in the ejection of droplets that have long filaments and satellite droplets.^62^ The calculated *Z* value for graphene ink was 4 (Table 1), indicating that the ink has suitable rheological properties for inkjet printing.

**Table 1:** Rheological Properties of the Graphene Ink

ρ (g/cm^3^)	σ (mN/m)	*d* (μm)	η (mPa·s)	*Z*
1	32	21.5	6.6	4

The contact angle of the graphene ink and PET substrate was measured to assess the wettability of the ink (Figure S3). A lower contact angle indicates better wetting,^63^ which enhances the adhesion of the ink to the substrate by promoting stronger intermolecular interactions. This improved wetting behavior is essential for achieving high print quality, as it ensures that ink droplets merge seamlessly, reducing defects such as gaps and irregular edges.^64^ The measured contact angle of 29° confirms the high wettability of the graphene ink on the PET substrate, facilitating high-quality printing.

To achieve successful inkjet printing without nozzle clogging, the size of the suspended particles in the ink must be at least 50 times smaller than the diameter of the printer nozzle.^65^ The dynamic light scattering (DSL) technique was utilized to measure the size of graphene particles in the ink employed for the development of graphene dry electrodes. As shown in Figure 2a, the DLS results indicate an average graphene particle size of 379.3 nm. Given that the nozzle diameter is 21.5 μm, these results confirm that the graphene particle size falls within the range suitable for printing.^74^

**Figure 2:**
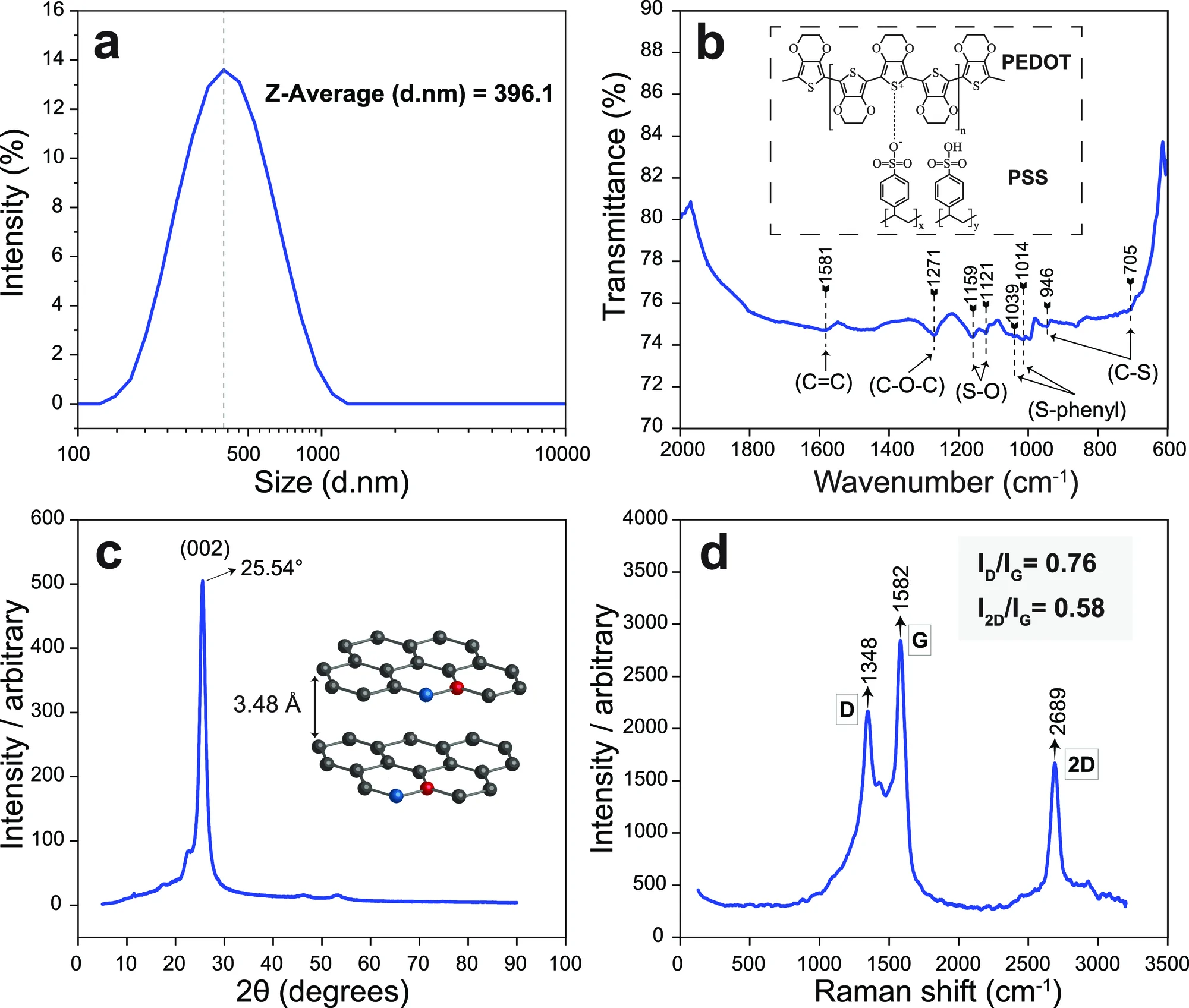
Material characterization of the printed dry graphene electrodes. (a) DLS measurement results of graphene ink. (b) FTIR spectra of graphene ink, including PEDOT:PSS precursor. (c) XRD results with interlayer spacing measurements. (d) Raman spectra showing intensity ratios of *I*_D_, *I*_G_, and *I*_2D_ graphene peaks.

The functional groups of the printed graphene electrodes with PEDOT:PSS precursor was characterized by Fourier transform infrared spectroscopy (FTIR), as shown in Figure 2b. The peaks at 1121, 1159, 1014, and 1039 cm^–1^ correspond to the S–O and S–phenyl bonds in sulfonic acid, respectively.^66^ Additionally, the C–S bonds in the thiophene backbone appear at 705 and 946 cm^–1^, while C–O–C stretching vibration peak is observed at 1271 cm^–1^ in the FTIR spectra.^67^ These bonds confirm the presence of PEDOT:PSS within the ink formulation, where it serves a dual role: the PSS component acts as a surfactant to ensure stable graphene dispersion and uniform film morphology, while the PEDOT chains facilitate electrical connectivity between individual graphene flakes. By acting as a conductive bridge, the PEDOT:PSS complex reduces the interflake contact resistance, thereby enhancing the overall electrical performance of the printed electrodes. Furthermore, the peak at 1581 cm^–1^ is indicative of the C=C aromatic stretch, which reflects the integrity of the sp^2^ honeycomb-like graphene structure.^68^

X-ray diffraction (XRD) analysis was carried out to investigate the crystallinity and interplanar spacing of the graphene sheets in the ink, as shown in Figure 2c. A diffraction peak at 25.54° was observed, corresponding to an interplanar spacing of 3.48 Å, with indices (002). This finding aligns with the characteristic diffraction peaks of multilayer graphene nanosheet.^69^

Raman spectroscopy was performed to analyze the defect intensity and the number of graphene layers. Figure 2d displays the Raman spectra, which feature three distinct peaks labeled D, G, and 2D, positioned at 1348, 1582, and 2680 cm^–1^, respectively. The G peak in the Raman spectrum of graphene corresponds to the high-frequency E_2g_ phonon at the Brillouin zone center. This peak results from the in-plane vibrations of sp^2^ carbon atoms and is a primary mode present in all sp^2^ carbon systems, providing essential information about the quality of the graphene.^70^ The D peak arises from the breathing modes of six-atom rings and is activated by defects. The presence and intensity of the D peak offer insights into the level of disorder or defects within the graphene lattice.^71^ The 2D peak is the second order of the D peak. Its shape and intensity are highly sensitive to the number of graphene layers, making it a valuable indicator for determining layer thickness.^72^ The *I*_D_/*I*_G_ ratio is useful for characterizing defect intensity. An obtained *I*_D_/*I*_G_ value of 0.76 suggests the presence of defects in the graphene lattice.^73^ Additionally, the intensity ratio of the 2D peak to the G peak (*I*_2D_/*I*_G_) can determine the number of layers in the graphene sample. An *I*_2D_/*I*_G_ value of 0.58 indicates a multilayer structure for the graphene.^74^

The cross-sectional profile and surface morphology of the printed graphene on PET substrates, with varying printing cycles, were characterized using Scanning Electron Microscopy (SEM). Figure 3a illustrates a uniform graphene coating with a thickness of 7.2 ± 0.3 μm after three printing cycles. The apparent intermixing at the interface is attributed to the thick hydrophilic coating of the commercial PET and mechanical artifacts introduced during manual sectioning with scissors, which distort the actual interface morphology. Figure 3b demonstrates significantly enhanced coverage and homogeneity as the printing cycles increased from one to three, which can directly result in improved electrical conductivity, thereby increasing the sensitivity for recording physiological signals through the skin.

**Figure 3:**
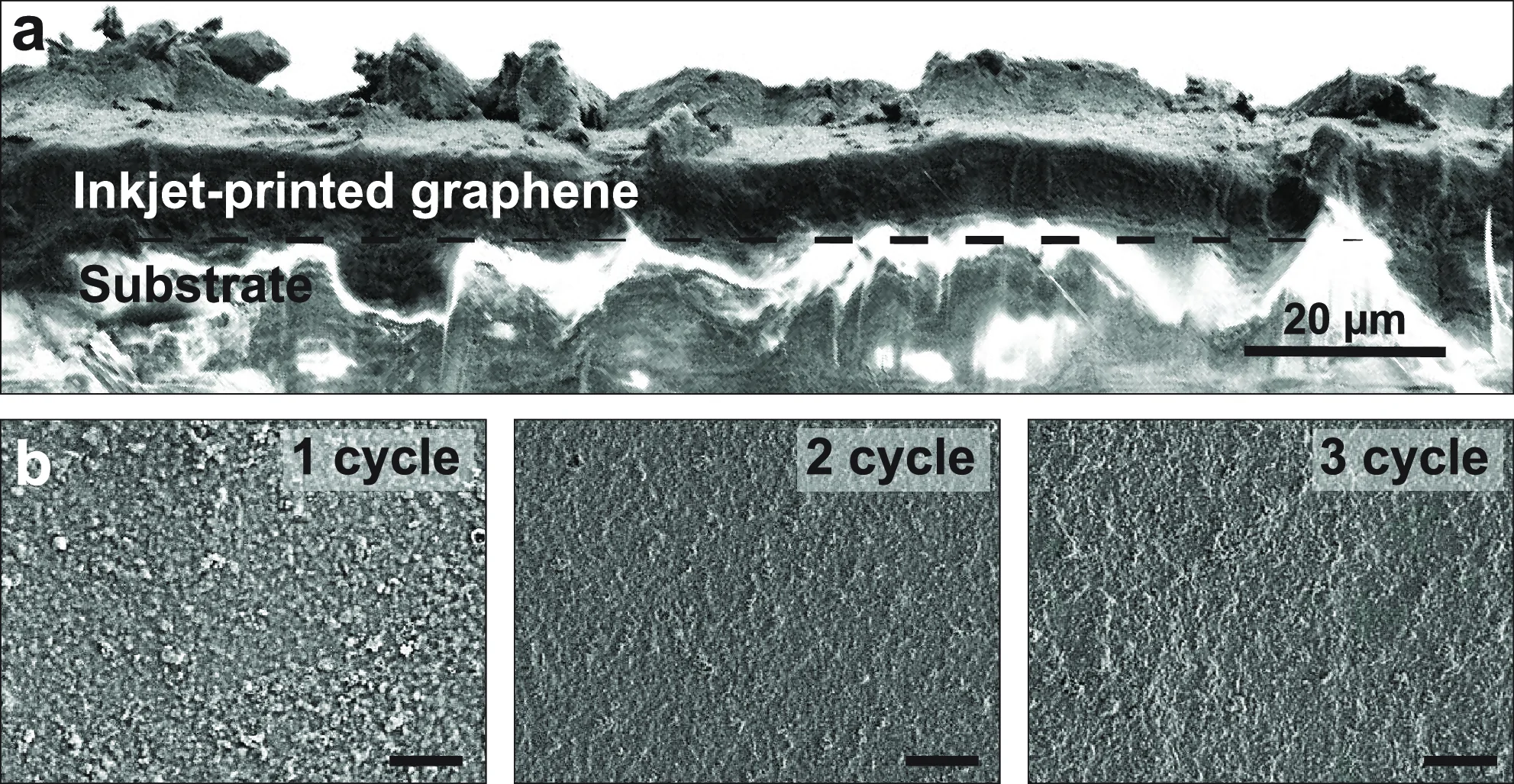
Cross-sectional profile and surface topography of inkjet-printed graphene electrodes on PET substrates. (a) Cross-sectional SEM image showing the surface topography and thickness of the printed graphene on the PET substrate. (b) Surface SEM images of printed graphene on PET substrates after 1, 2, and 3 printing cycles.

### 2.3 Electrical Characterization

Figure 4a represents the sheet resistance (Ω/sq) values of inkjet-printed graphene samples based on the number of printing cycles over a period of 15 days. It is evident that the sheet resistance is significantly influenced by the number of inkjet-printed cycles, with electrical conductivity improving as the number of cycles increases and consequently, the thickness of the deposited graphene also increases. The sheet resistance values were 352.6 (Ω/sq) for 1 cycle, 202.3 (Ω/sq) for 2 cycles, and 79.7 (Ω/sq) for 3 printing cycles. The sheet resistance values relatively increased over the case of 15 days for roughly 11% for 1, 2, and 3 cycle inkjet-printed graphene electrodes. In the inkjet-printed silver nanoparticle samples, the sheet resistance values were measured as 41.3 (Ω/sq) for 1 cycle, 7.4 (Ω/sq) for 2 cycles, and 1.2 (Ω/sq) for 3 printing cycles.

**Figure 4:**
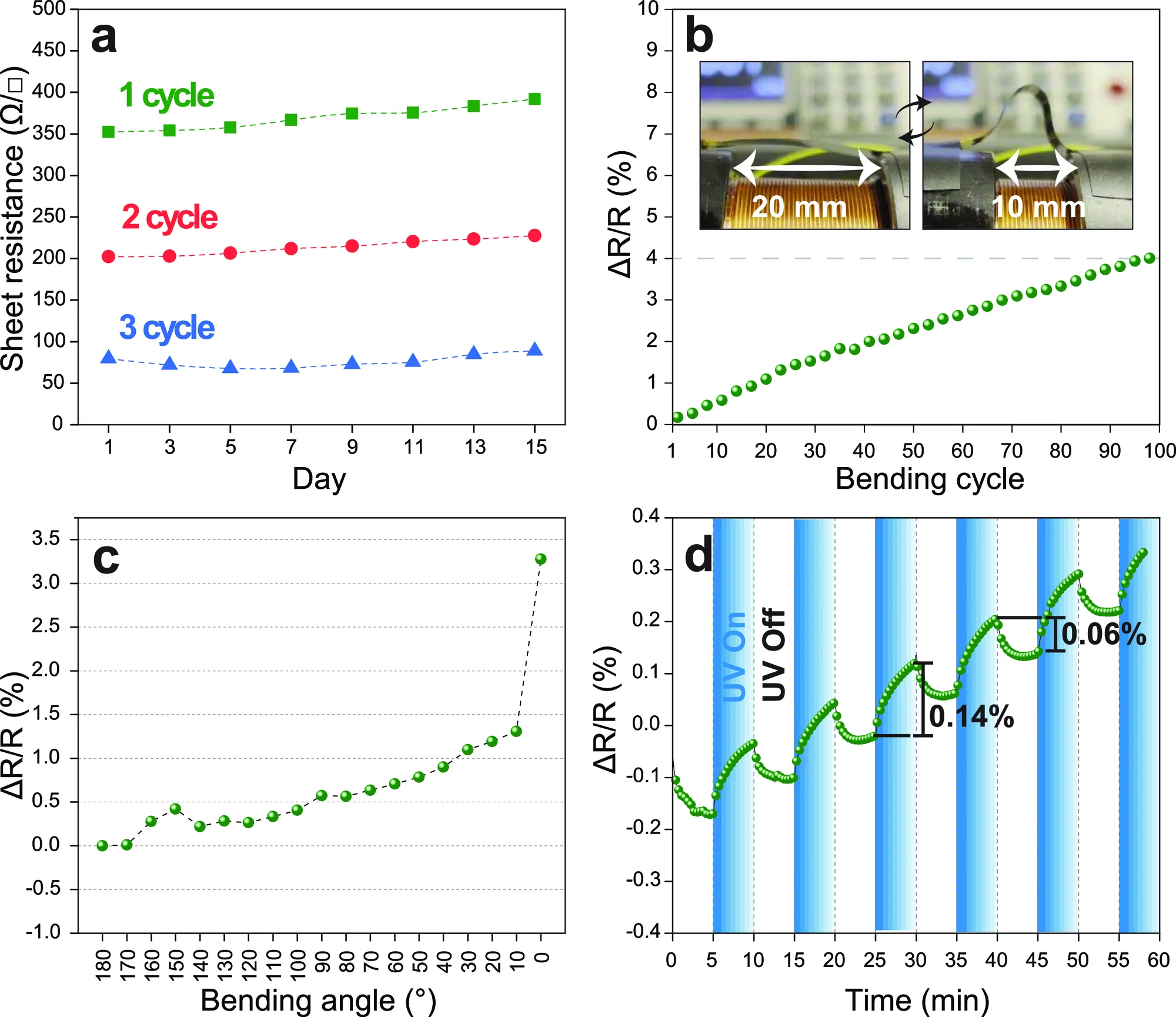
Electrical characterization of the printed graphene electrodes. (a) Sheet resistance variations of 1, 2, and 3 cycle printed graphene electrodes over a 15-day period. (b) Resistance variations of printed graphene electrodes during 100 cyclic bending cycles, with the inset showing the experiment setup. (c) Resistance variations under different bending angles. (d) Resistance variations under cyclic UV light exposures.

The flexibility of the printed graphene electrodes was evaluated by measuring resistance variations during mechanical deformations, particularly bending. The results shown in Figure 4b and Movie S2 demonstrate a mere 4% increase in resistance after 100 cyclic bending, indicating that mechanical deformations have an insignificant impact on the electrical conductivity of the electrodes and, consequently, their efficacy in recording physiological signals. Moreover, Figure 4c presents the change in electrical resistance as a function of bending angle. The data reveal a minimal resistance change of approximately 0.077% for every 5° of bending. Additionally, the observed resistance increase of only 3.27% under complete folding further highlights the exceptional flexibility of the inkjet-printed graphene electrodes.

The resistance variations of inkjet-printed graphene under ultraviolet (UV) light with 405 nm emission wavelength were analyzed to evaluate the performance of the electrodes when exposed to intense UV radiation, such as sunlight. Figure 4d presents the results of periodic 5 min UV exposures over a total duration of 1 h. The findings indicate that the printed graphene electrodes experienced a resistance shift of +0.14% during UV exposure followed by a −0.06% shift when the UV source was turned off, each measured over 5 min. Additionally, the baseline resistance increased by 0.3% by the end of the experiment. This phenomenon can be attributed to oxygen molecules from the ambient air adsorbing onto the defects and edges of the graphene, which leads to hole doping. When illuminated by UV light, photoinduced plasmon excitation disrupts the oxygen-graphene binding, resulting in the desorption of oxygen molecules. This process reduces the hole concentration in the graphene, thereby increasing its electrical resistance.^75^ Notably, these findings suggest that while graphene is a UV-responsive material, the 0.3% shift in the electrical resistance represents a transient surface effect rather than irreversible structural aging. This minimal baseline drift, coupled with the high input impedance of acquisition electronics, ensures that long-term environmental exposure does not compromise the fidelity of electrophysiological recordings. Furthermore, these results indicate that inkjet-printed graphene may be a viable option for flexible sensors designed for UV detection.

The adhesion quality of the printed graphene ink to the PET substrates was assessed using a qualitative Scotch-tape peel test (Movie S3).^76^ The experiment involved applying the sticky side of the tape to an inkjet-printed graphene sample, pressing the tape against the sample, and gently peeling the tape off the sample. The test was repeated 4 times and the resistance of the sample was measured during the process. No graphene particles were visually observed on the tape after the tests. Additionally, the resistance of the printed graphene sample remained unchanged before and after the experiment. These results indicate excellent adhesion of the printed graphene to the substrates, making them ideal candidates for repeated use over extended periods and intuitive to don and doff.

### 2.4 Biopotential Data Collection Using Printed Electronics

The dry inkjet-printed electrodes demonstrate effective recording capabilities for various biopotential signals. Figure 5a illustrates the placement of these electrodes for recording ECG signals in the Lead-I configuration, with two electrodes positioned on the left and right arms. Figure 5b presents simultaneous recordings of ECG signals from printed graphene, silver nanoparticle (AgNP), and commercial Ag/AgCl electrodes. The clearly identifiable PQRST segments observed in both the graphene and AgNP-based electrodes, compared to the gel-based commercial Ag/AgCl electrodes, confirm their effectiveness in recording ECG signals. Furthermore, the printed graphene and AgNP electrodes exhibit correlation ratios of 99.34% and 99.48%, respectively, when compared to the Ag/AgCl electrodes. These high correlation ratios indicate that the printed electrodes are capable of providing accurate and consistent biopotential signal recordings, comparable to the established commercial electrodes.

**Figure 5:**
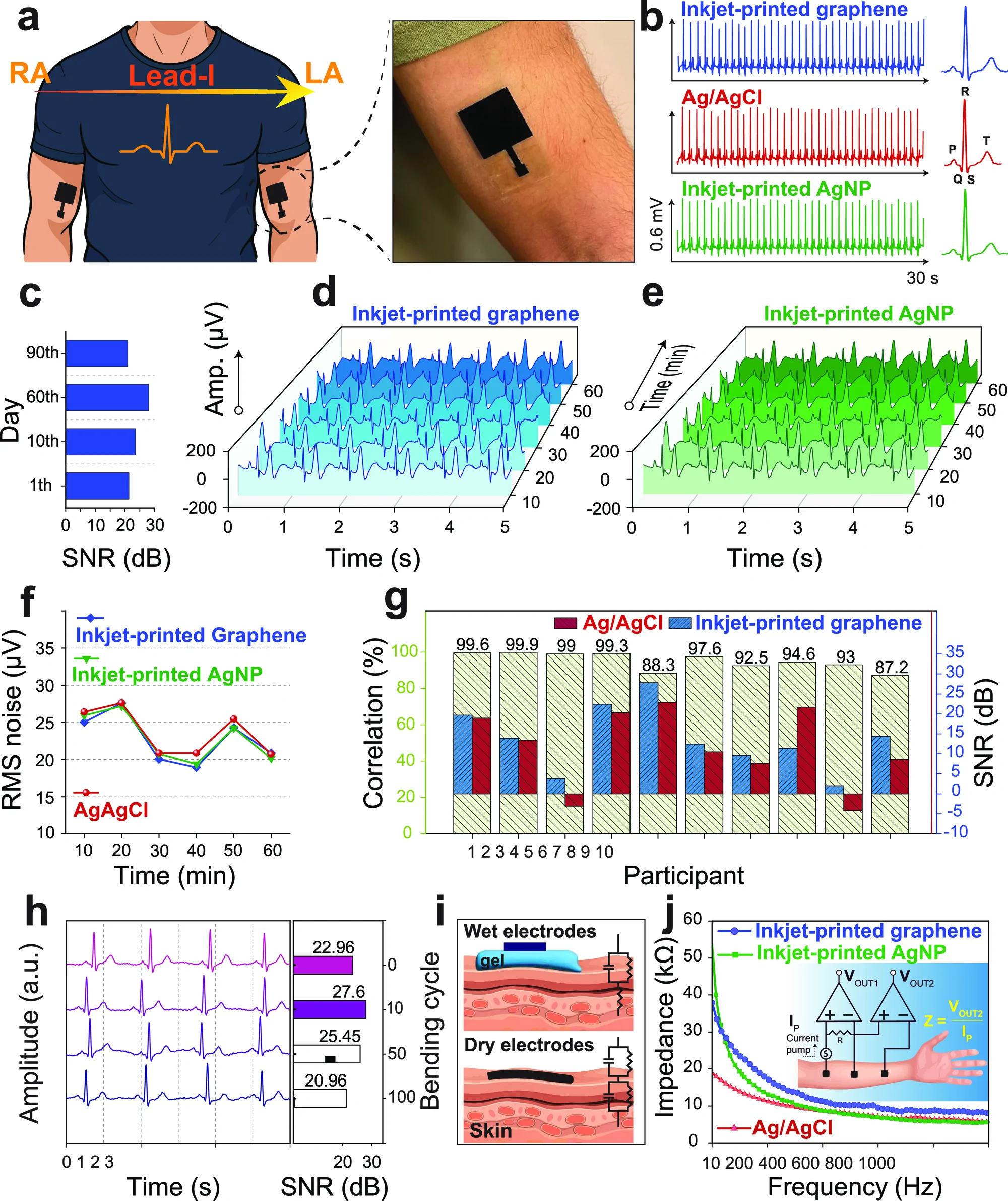
Overview of inkjet-printed dry electrodes in recording electrocardiography (ECG) signals. (a) Placement of dry inkjet-printed electrodes in the Lead-I configuration on the left and right arms. (b) Simultaneous ECG recordings from printed graphene, AgNP, and commercial Ag/AgCl electrodes. (c) Signal-to-noise ratio (SNR) values for ECG signals recorded on the first, tenth, sixtieth, and ninetieth days after printing. (d) Continuous ECG signal recording over 60 min using printed graphene and AgNP electrodes. (e) RMS noise levels for printed graphene and AgNP electrodes over a 1 h recording period. (f) ECG signal recordings from a diverse group of ten participants, showing correlation ratios and SNR values for printed graphene and commercial Ag/AgCl electrodes. (g) Performance of printed graphene electrodes before and after 10, 50, and 100 cyclic bending cycles. (h) Impedance modeling at the skin-electrode interface for gel-based and gel-free dry electrodes. (i) Skin-electrode impedance values for printed graphene, AgNP, and Ag/AgCl electrodes.

One significant advantage of the printed graphene electrodes is their long shelf life and reusability. This addresses the common limitation of commercial biopotential monitoring with Ag/AgCl electrodes, where gel dehydration can reduce their reusability over time. The signal-to-noise ratio (SNR) values of 21.3, 23.5, 28.0, and 20.9 dB for the recorded ECG signals collected on the first, tenth, sixtieth, and ninetieth days after printing demonstrate their reusability without performance loss (Figure 5c). Additionally, this advantage positions them as remarkable candidates for use in low-income and resource-deprived regions, where the continuous replacement of commercial electrodes would impose a significant financial burden.

The printed electrodes enable continuous biopotential signal recording over extended periods. Figure 5d,e demonstrate the recording of ECG signals over 60 min using the printed graphene and AgNP electrodes, with no signal degradation observed over time. To quantitatively assess the performance of the electrodes during the 1 h signal recording, root-mean-square (RMS) noise evaluation was performed. RMS noise is an indicator of signal fluctuations,^77^ which can originate from disruptions at the skin-electrode interface and is indicative of the quality of signal collection.^78^ The results show that, over the 1 h period, both the printed graphene and AgNP electrodes exhibited noise levels comparable to those of clinical-grade gel-based Ag/AgCl electrodes (Figure 5f). Specifically, the RMS noise levels for the graphene electrodes varied from 32.59 μV after 10 min of ECG recording to 19.75 μV at the end of 1 h. A similar trend was observed for the printed AgNP electrodes, with RMS noise decreasing from 26.66 μV to 16.87 μV after 1 h of signal recording. These reported RMS noise values indicate higher signal quality than previously reported for inkjet-printed dry electrodes.^79^

To ensure the robustness and generalizability of the printed graphene dry electrodes, ECG signal recordings were conducted on a diverse group of ten participants. This group included individuals with varying body mass indices (BMIs), age, and comprised both men and women (Table S2). As shown in Figure 5g, the recorded ECG signals from the participants revealed a correlation ratio of 95.14 ± 4.75% between printed graphene and clinical grade standard Ag/AgCl electrodes. Additionally, the SNR analysis indicated 13.74 ± 7.97 dB for the printed graphene electrodes and 11.65 ± 9.73 dB for the commercial Ag/AgCl electrodes. The slightly higher SNR values reported for printed graphene electrodes compared to Ag/AgCl electrodes can be attributed to the larger surface area of printed graphene electrodes (5.5 cm^2^) interfacing the skin compared to the circular active gel area in commercial Ag/AgCl electrodes (2.15 cm^2^).^80^ This phenomenon allows for more efficient charge transfer between the skin and the electrode, leading to higher SNR values. The cumulative results of the correlation ratio and SNR values of the ECG data collected from a heterogeneous participant pool of varying BMIs, genders, and ages demonstrate the effective functionality of the printed graphene electrodes across a wide range of physiological conditions, ensuring they are suitable for clinical applications.

The performance of the printed graphene electrodes was evaluated before and after subjecting them to 10, 50, and 100 cyclic bending cycles (Figure 5h). The recorded ECG signals showed clear PQRST segments after each bending trial, confirming that the printed electrodes have minimal sensitivity to mechanical deformations. Furthermore, the SNR values were 22.96 dB before bending and 20.06 dB after 100 bending cycles, demonstrating the durability and flexibility of the printed graphene electrodes. This resilience significantly contributes to their ability to withstand various movements and stresses, making them suitable for practical applications.

Skin-electrode impedance is a critical factor in recording biopotential signals, as the integrity and quality of these signals depend highly on it. Figure 5i illustrates the impedance modeling at the skin-electrode interface for both gel-based and gel-free dry electrodes. The major difference arises from the absence of a gel medium in dry electrodes, leading to a capacitive effect between the electrode and the skin. This effect can be modeled as a parallel capacitor element, which, depending on ambient conditions such as skin moisture and the pressure applied to the electrode, can significantly influence the overall skin-electrode impedance.^39^

The skin-electrode impedance was measured using a previously reported method, as shown in the inset of Figure 5j.^49^ The impedance values for the printed graphene, AgNP, and Ag/AgCl electrodes were 37.23 kΩ, 53.39 kΩ, and 18.83 kΩ at 10 Hz, respectively. These results demonstrate that the reported dry electrodes can effectively collect physiological signals, with skin-electrode impedance values comparable to those of gel-based gold-standard Ag/AgCl electrodes. The relatively low impedance of the graphene electrodes, even in the absence of a conductive gel, may be further attributed to the PEDOT:PSS within the ink formulation. By acting as a secondary conductive phase that bridges the graphene flakes, PEDOT:PSS facilitates a more continuous percolation network, effectively lowering the internal resistance of the electrode and promoting efficient charge transfer at the skin-electrode interface.

Electroencephalography (EEG) signals were collected simultaneously using printed graphene, AgNP, and commercial Ag/AgCl electrodes to assess their performance in recording highly noise-susceptible signals with amplitudes in the microvolt range. The EEG signals were recorded in a unipolar referential montage using the universal standardized 10–20 system. Inkjet-printed AgNP, commercial Ag/AgCl, and inkjet-printed graphene electrodes were placed on the Fp1, Fpz, and Fp2 regions, respectively. Figure 6a illustrates the collected EEG signals over a 120-s period, where the first 60 s involved the participant looking forward and performing voluntary eye blinks. The observed amplitude spikes correspond to random and voluntary eye blinks. These blinks disappeared from the signal when the participant closed their eyes in a relaxed position during the second 60 s of the experiment. Figure 6b shows the power spectral density (PSD) of the recorded EEG signals during the two experimental phases of open and closed eyes. The observed peaks around 10 Hz in the closed-eye phase can be attributed to α waves, which lie within the 8–13 Hz range. These waves are associated with relaxed awareness of mental states, excluding any attention.^30^ The observed α frequencies exhibited approximately 200 μV^2^/Hz power densities for the printed graphene, AgNP, and commercial gel-based Ag/AgCl electrodes. The results clearly demonstrate that the reported printed dry electrodes possess significant capabilities in recording ultraweak neural signals from the skin, with performance comparable to gel-based Ag/AgCl electrodes.

**Figure 6:**
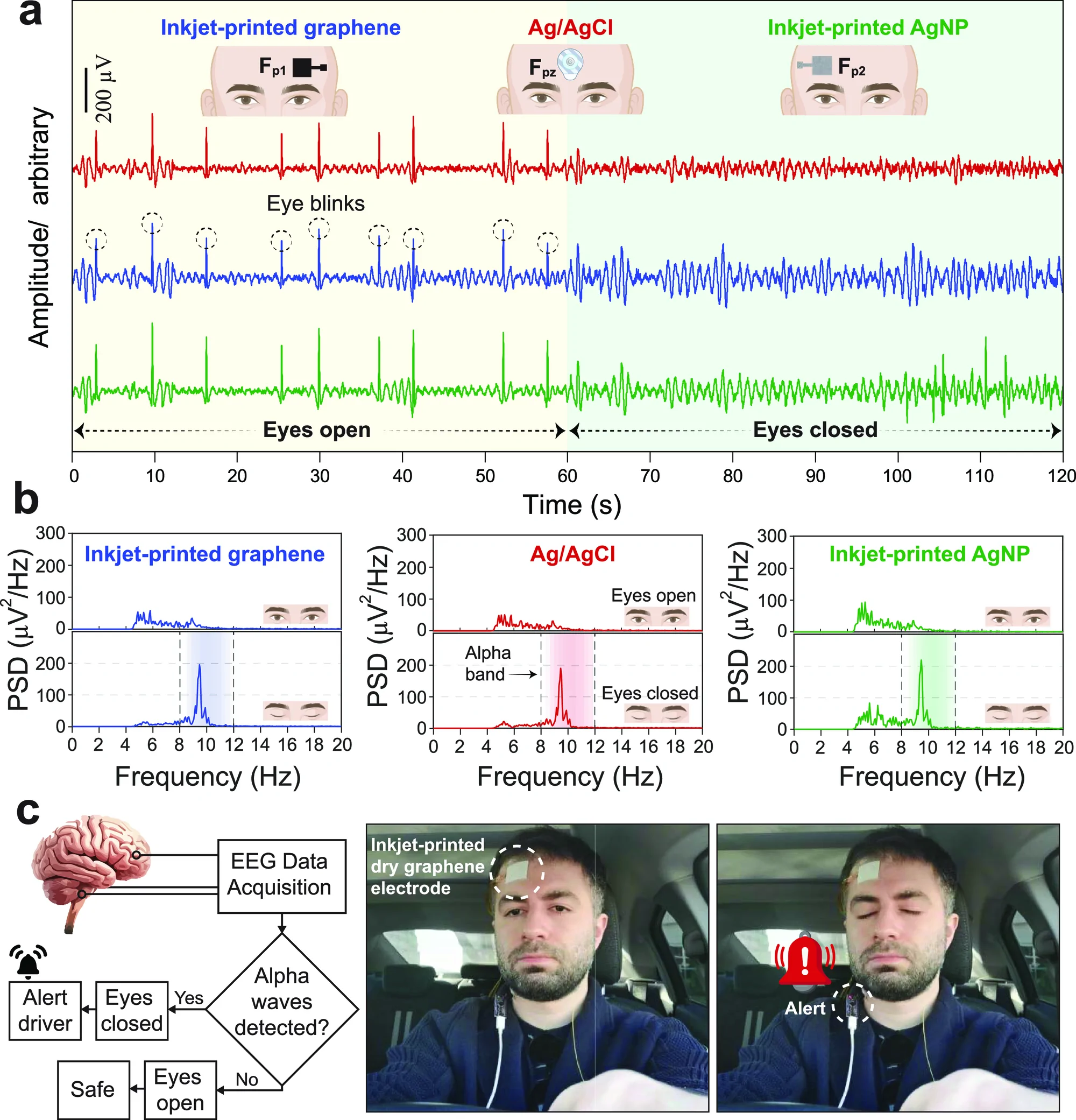
EEG signal recording with printed dry electrodes. (a) Collected EEG signals over a 120-period with schematic illustration of electrode placement. (b) PSD of recorded EEG signals during open and closed eye phases, with observed α wave peaks around 10 Hz in the closed-eye phase. (c) Integrating printed graphene electrodes for drowsiness monitoring in drivers.

A novel application of continuous monitoring using wearable devices can be the detection of drowsiness in car drivers through EEG signals, alerting the driver to prevent fatal accidents. While image processing tools can monitor facial changes, such as whether the eyes are open or closed, their dependence on high-quality images and adequate environmental lighting limits their effectiveness.^81^ Many car accidents caused by drowsiness occur at night under poor lighting conditions, further highlighting the limitations of camera-based detection systems in accurately tracking eye states. Figure 6c illustrates the use of inkjet-printed graphene electrodes for continuous EEG signal recording to detect eye states and drowsiness. The presented system relies on detecting α waves generated a few seconds after the transition from open to closed eyes. By precisely identifying α frequencies in EEG signals, the proposed system provides auditory stimuli to alert the driver in cases of drowsiness, waking them up to avoid fatal accidents (Movie S4). These results underscore the effectiveness of printed graphene electrodes in integrating clinical physiological signals into daily wearable applications without relying on conspicuous clinical instrumentations.

To further evaluate the efficacy of the printed electrodes, electrooculography (EOG) signals were asynchronously collected using printed graphene, AgNP, and Ag/AgCl electrodes to assess their performance in capturing eye movement data. EOG signals can be used to develop advanced human-computer interfaces. For instance, wearable EOG devices can enable eye-based control of computers and other devices, making technology more accessible for individuals with physical disabilities.^36^ These signals are generated due to the potential difference between the cornea and the retina. EOG experiments involve positioning two electrodes on the right and left temples, with a reference electrode on the middle part of the forehead. The experiment involved a participant in front of a predetermined center point, with two marks positioned approximately 65 cm to the right and left of the center point. The participant was instructed not to talk or move to avoid generating undesired facial electromyography (EMG) and motion artifacts. To generate EOG signals, the participant was asked to look to the left (left gaze), wait for 10 s, and then look forward with voluntary eye blinks (forward gaze). This was followed by a right gaze for the same duration. Figure 7a presents the collected EOG biopotentials, showing unique EOG patterns resulting from horizontal saccades and random blinks, collected from printed graphene, AgNP, and conventional wet electrodes.

**Figure 7:**
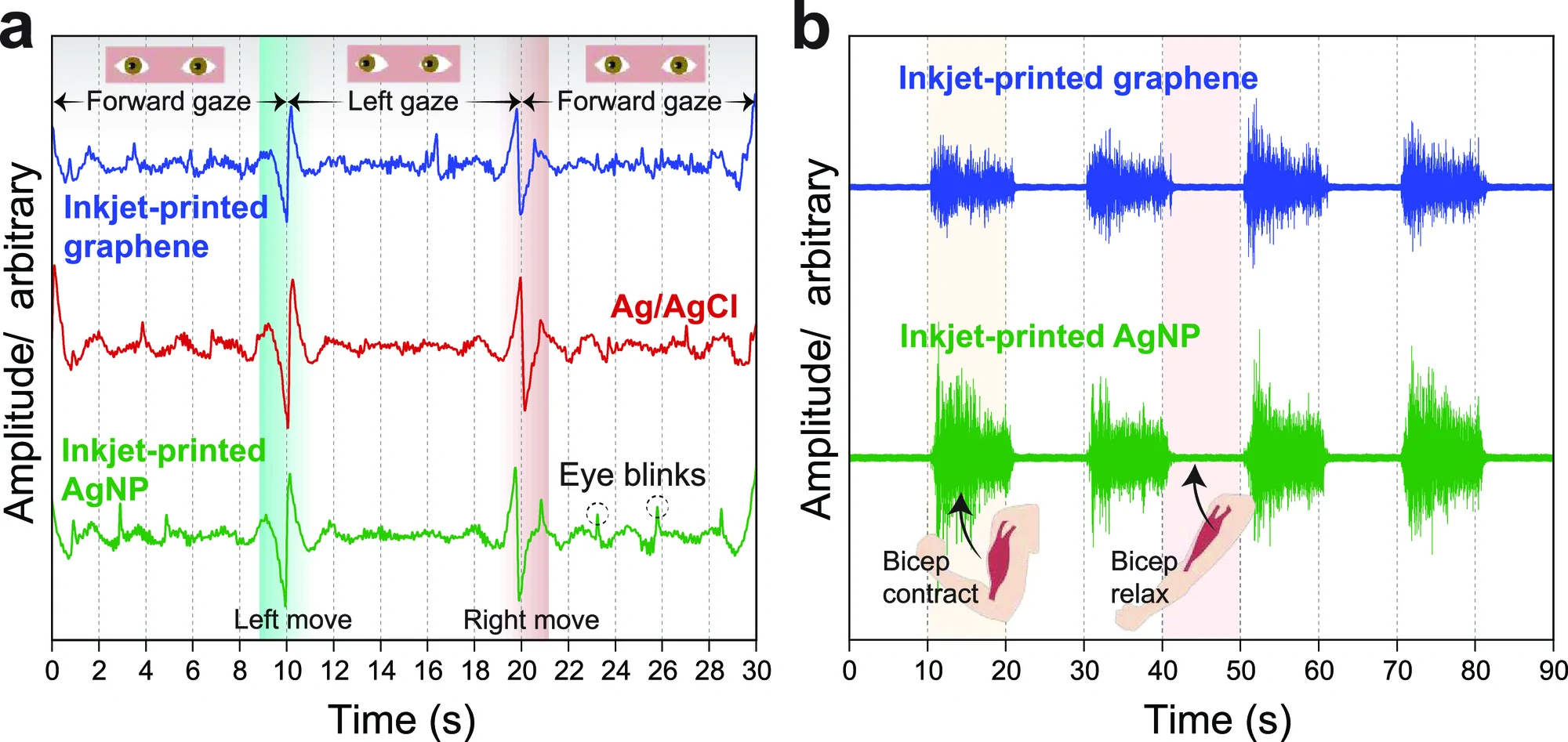
EOG and EMG signal recording with printed electrodes. (a) Collected EOG biopotentials showing unique patterns from horizontal saccades and random blinks, using printed graphene, AgNP, and conventional wet Ag/AgCl electrodes. (b) EMG signals recorded synchronously from inkjet-printed graphene and AgNP electrodes in a bipolar configuration for the right-hand biceps brachii during contraction and relaxation phases.

Unlike other physiological biopotentials, EMG signals are more susceptible to motion artifacts. This is because experiments examining a specific muscle group using EMG data usually involve the physical motion of the muscle under study. Therefore, electrode conformability plays a crucial role in signal quality when recording EMG signals. Figure 7b demonstrates the EMG signals recorded synchronously from inkjet-printed graphene and AgNP electrodes in a bipolar electrode configuration for the right-hand biceps brachii while the participant performed a biceps contraction and relaxation every 10 s. The acquired EMG signals show distinguishable patterns in different phases of contraction and relaxation. To quantitatively assess the recorded signals from printed AgNP and graphene electrodes, the area under the curve (AUC) between the upper and lower envelopes of four consecutive EMG signals during the contraction phase was calculated. The results indicated an AUC of 6698 ± 825 μV·s for the printed graphene and 9716 ± 1240 μV·s for the printed AgNP electrodes. The higher AUC observed for the AgNP electrodes can be attributed to their relatively higher electrical conductivity, which enhances signal transmission, leading to stronger EMG signals and higher AUC values. However, both the AgNP and graphene electrodes demonstrated significant capabilities in capturing EMG signals, indicating their effectiveness in recording muscle activity. This suggests that both types of electrodes are suitable for EMG applications, providing reliable and high-quality signal recordings.

## 3 Conclusions

In summary, this work presents gel-free and reusable flexible dry electrodes, achieved by precisely tuning and optimizing the inkjet printing process for fabricating graphene-based electrophysiological electrodes. The inkjet-printed graphene electrodes on PET substrates, when interfaced with the skin in precise clinical configurations, demonstrated excellent performance in recording ECG, EEG, EMG, and EOG signals. Quantitative assessments of the recorded ECG signals showed a 99.34% correlation ratio between the signals collected from printed graphene and commercial Ag/AgCl electrodes, with a similar score of 99.48% obtained when benchmarking printed AgNP electrodes against Ag/AgCl electrodes. A comparable correlation of 95.14 ± 4.75% was observed in 10 participants with varying demographic markers. Additionally, the printed graphene electrodes exhibited high endurance under 100 cyclic bends and strong bonding between the graphene and the substrate, as verified through Scotch tape experiments, highlighting their excellent flexibility and reusability. Furthermore, the applicability of the proposed dry electrodes in real-life wearable systems was demonstrated by accurately collecting and characterizing neural signals for detecting drowsiness in car drivers, thereby contributing to accident prevention.

## 4 Experimental Section

### 4.1 Materials

A commercially available water-based graphene ink (DM GRA-9003) containing poly­(3,4-ethylenedioxythiophene)-polystyrene sulfate (PEDOT:PSS) as a surfactant and dilution solvent was purchased from Dycotec Materials Ltd. (UK). Silver nanoparticle ink (AgNP) with 40% silver concentration, 8–12 cP viscosity, 30–50 nm particle size, and 28–32 mN/m surface tension was purchased from NovaCentrix (Metalon JS-A191). A poly­(ethylene terephthalate) (PET) film, 135 μm thick and coated with a hydrophilic material (Hp Premium Inkjet Transparent Film), was used as the substrate.

### 4.2 Ink Conditioning

The purchased graphene ink (viscosity: >8 cP and surface tension: 24–30 mN/m) was diluted in an aqueous solvent containing diethylene glycol and ethanol to achieve the required viscosity for the inkjet printing process, resulting in a viscosity of 6.6 cP and surface tension of 32.9 mN/m at 25 °C. To obtain a homogeneous dispersion and prevent agglomeration, the ink was stirred at 300 rpm for 15 min, followed by probe sonication for 30 min, and degassed prior to printing.

### 4.3 Material Characterization

Contact angle measurements were performed using the sessile drop method by placing a drop of graphene ink onto a PET substrate. The side-view profile of the droplet-substrate interface was captured, and the contact angle was measured 10 s after drop formation (Theta Lite optical tensiometer, ON, Canada). Dynamic light scattering (DLS) and Zeta (ζ) potential data were collected using a Malvern Zetasizer, with the graphene dispersion diluted at a volumetric ratio of 1:2000 before testing. Scanning electron microscopy (SEM) (Zeiss Gemini Supra 35 VP, LEO, Germany) was conducted at an acceleration voltage of 3 kV. Cross-sectional samples were prepared by manually sectioning the inkjet-printed graphene electrodes using stainless steel scissors. X-ray diffraction (XRD) analysis was performed within the 2θ range of 5° to 90°, using a scanning rate of 3° per minute with Cu Kα radiation (Bruker D8 Advance instrument, Germany). The parameters were set to an X-ray beam wavelength of 1.5418 Å, a voltage of 40 kV, and a current of 40 mA. Fourier-transform infrared spectroscopy (FTIR, Thermo Scientific, Nicolet iS10) was used to investigate the chemical composition of inkjet-printed samples. Raman spectra of the graphene inkjet-printed PET substrate were recorded using a Raman spectrometer (Renishaw inVia Reflex Raman Microscope and Spectrometer) with an excitation wavelength of 532 nm. Sheet resistance measurements were assessed using the four-probe technique (Cascade Microtech CP4).

### 4.4 Inkjet Printing

Inkjet printing was performed using a Fujifilm Dimatix (DMP-2850) inkjet printer (Fujifilm, Santa Clara, CA, USA) with a loaded Dimatix materials cartridge (Samba Dimatix, Fujifilm, Santa Clara, CA, USA). The cartridge head incorporates 12 piezoelectric jetting nozzles, each with a diameter of less than 22 μm and capable of jetting a 2.4 pL drop volume. The graphene electrodes were printed by applying a desired pressure through a unipolar customized waveform of four successive square pulses of 1.8 μs, voltage of 27–33 V, drop velocity of 4 m/s, jetting frequency of 2.5 kHz, and 10 μm drop spacing. Waveform manipulation was performed to accommodate the increased drop volume relative to the standard Fujifilm Model Fluid waveform. Additionally, the selected frequencies ensured precise drop placement on the substrate, enabling high-quality printing with minimal offset. The printed graphene electrodes were annealed at 120 °C for 60 min. For the printed AgNP electrodes, the original Fujifilm Model Fluid waveform was applied to the piezoelectric elements with a voltage of 27–33 V, drop velocity of 7 m/s, jetting frequency of 10 kHz, and 10 μm drop spacing, followed by a postannealing step at 140 °C for 10 min. Both the graphene and AgNP electrodes were printed for three cycles with 60 s intervals between each printing cycle. Additionally, the printer platen temperature was set to 40 °C for graphene and 30 °C for AgNP to evaporate the solvents during printing.

### 4.5 Data Collection and Signal Analysis

Physiological biopotential signals of voluntary participants were collected with their informed consent and by following the guidelines of the Helsinki Declaration of 1964, as revised in 2013, and approved by the ethics committee of Sabanci University (FENS-2020–48). For data acquisition, an open-source unit (Cyton Board, OpenBCI) with a built-in 24-bit Delta-sigma analog-to-digital converter was used. Signal-to-noise ratio (SNR) and RMS noise analysis were performed to evaluate the collected ECG signal quality using a custom script in MATLAB (Mathworks, USA). The energy around the QRS complex was defined as the “signal,” while the remaining baseline fluctuations between the R peaks, including P- and T-waves and the isopotential line, were defined as “noise.”

2 $$\mathrm{SNR}\;\left[\mathrm{dB}\right]=20\log\left(\frac{\left|E_{\mathrm{rms},\mathrm{QRS}}\right|}{E_{\mathrm{noise}}}\right)$$

## Supplementary Material











## Data Availability

All data utilized in the preparation of this manuscript are included within the main text or the Supporting Information. Any additional data may be provided by the authors upon reasonable request.

## Notes

Jetting profile of the graphene ink; Zeta potential measurement of graphene ink; contact angle measurement of graphene ink on PET substrate; comparison of inkjet-printed graphene electrodes and state-of-the-art sensors; demographic characteristics of ten participants for ECG signal recordings (PDF)
Jetting profile of graphene inkjet printing (Movie S1) (MP4)
Resistance stability of printed graphene electrodes under cyclic bending (Movie S2) (MP4)
Adhesion analysis of inkjet-printed graphene on pet using scotch tape experiment and resistance measurement (Movie S3) (MP4)
EEG safety system to detect and alert drowsy drivers (Movie S4) (MP4)
