# Supplementary material for: Inkjet-Printed Aqueous Graphene Suspensions on Flexible Substrates for Multimodal Wearable Continuous Health Monitoring
Source: ACS Nanosci Au. 2026 Apr 22;6(4):582–95. doi: 10.1021/acsnanoscienceau.5c00195 (PMC13495504; doi:10.1021/acsnanoscienceau.5c00195)
Supplement: Supplementary file 1 [file ng5c00195_si_001.pdf]

Supporting Information

Inkjet-printed Aqueous Graphene Suspensions on Flexible Substrates for Multimodal Wearable Continuous Health Monitoring

Seyed Sajjad Mirbakht [1,2], *Faruk Ballipinar* [1,2,3], *Burcu Arman Kuzubasoglu* [1,2,3], *Melih Can Tasdelen* [1,2], *Saygun Guler* [1,2], *Murat Kaya Yapici* [1,2,3,4*]

[1] Faculty of Engineering and Natural Sciences, Sabanci University, 34956 Istanbul, Türkiye.

[2] Sabanci University Micro/Nano Devices and Systems Lab (SU-MEMS), Sabanci University, 34956 Istanbul, Türkiye.

[3] Sabanci University SUNUM Nanotechnology Research Center, 34956 Istanbul, Türkiye.

[4] Department of Electrical and Computer Engineering, University of Washington, 98195 Seattle, USA.

[*] Corresponding author E-mail: murat.yapici@sabanciuniv.edu

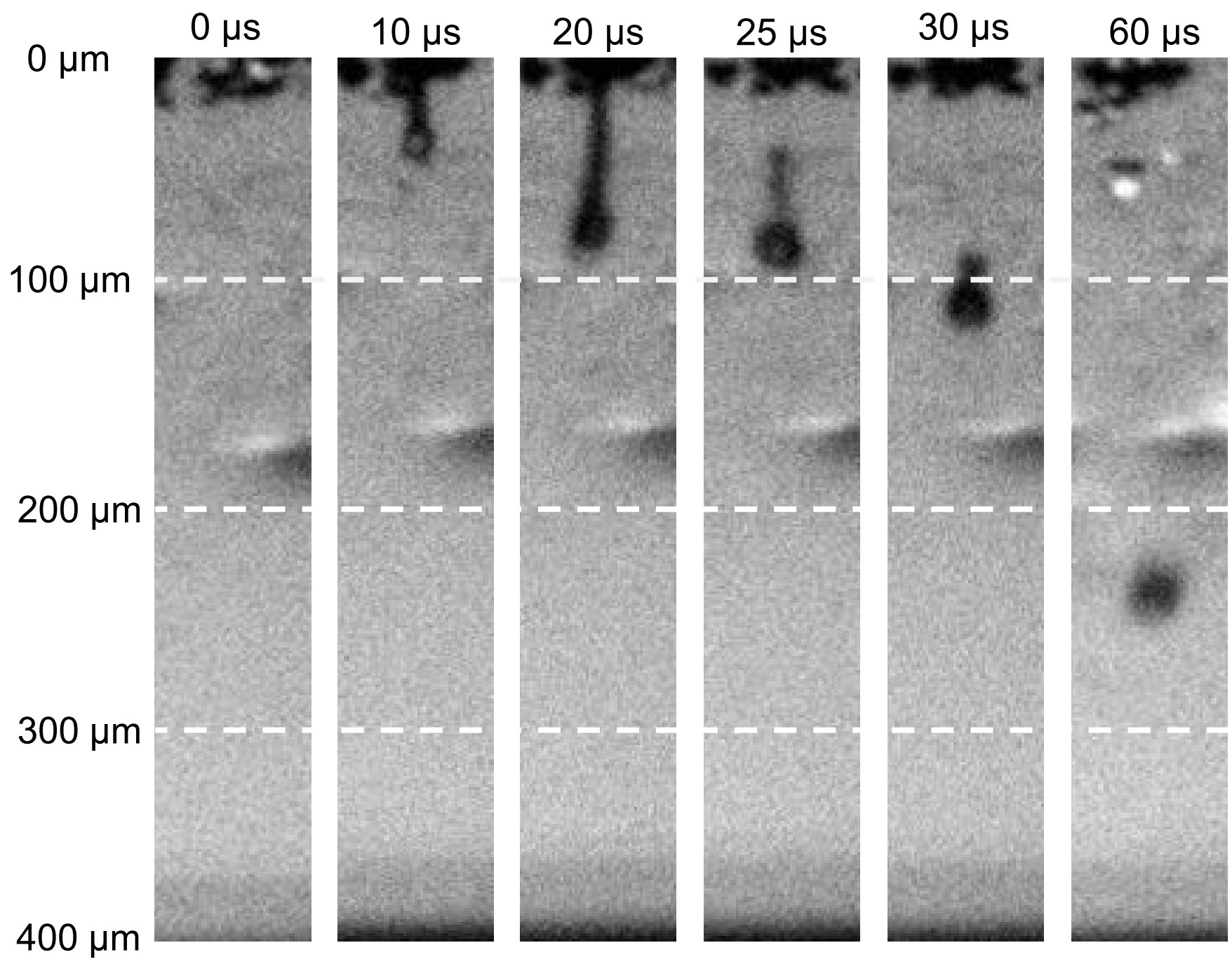

Figure S1. Jetting profile of the graphene ink: a drop displacement of ~240 μm in 60 μs yields a drop velocity of 4 m/s

Zeta potential (mV)	Std. Deviation (mV)	Conductivity (mS/cm)	Effective voltage (V)	Count rate (kcps)
-16.3	3.56	0.419	151.7	61.5

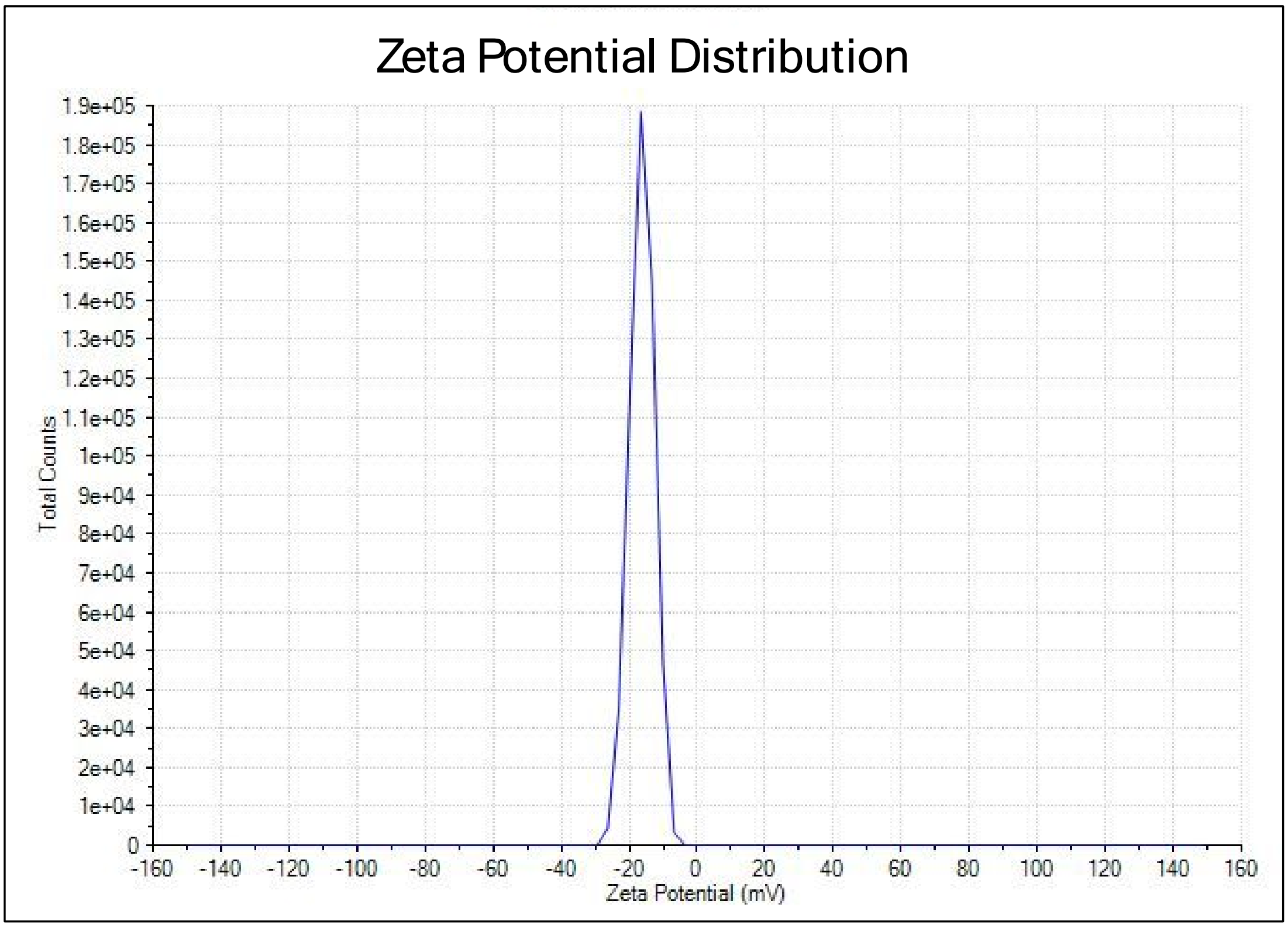

Figure S2. Zeta Potential Measurement of Graphene Ink. The zeta potential measurement of the graphene ink is measured as -16.3 ± 3.56 mV, indicating that the graphene dispersion is relatively stable.

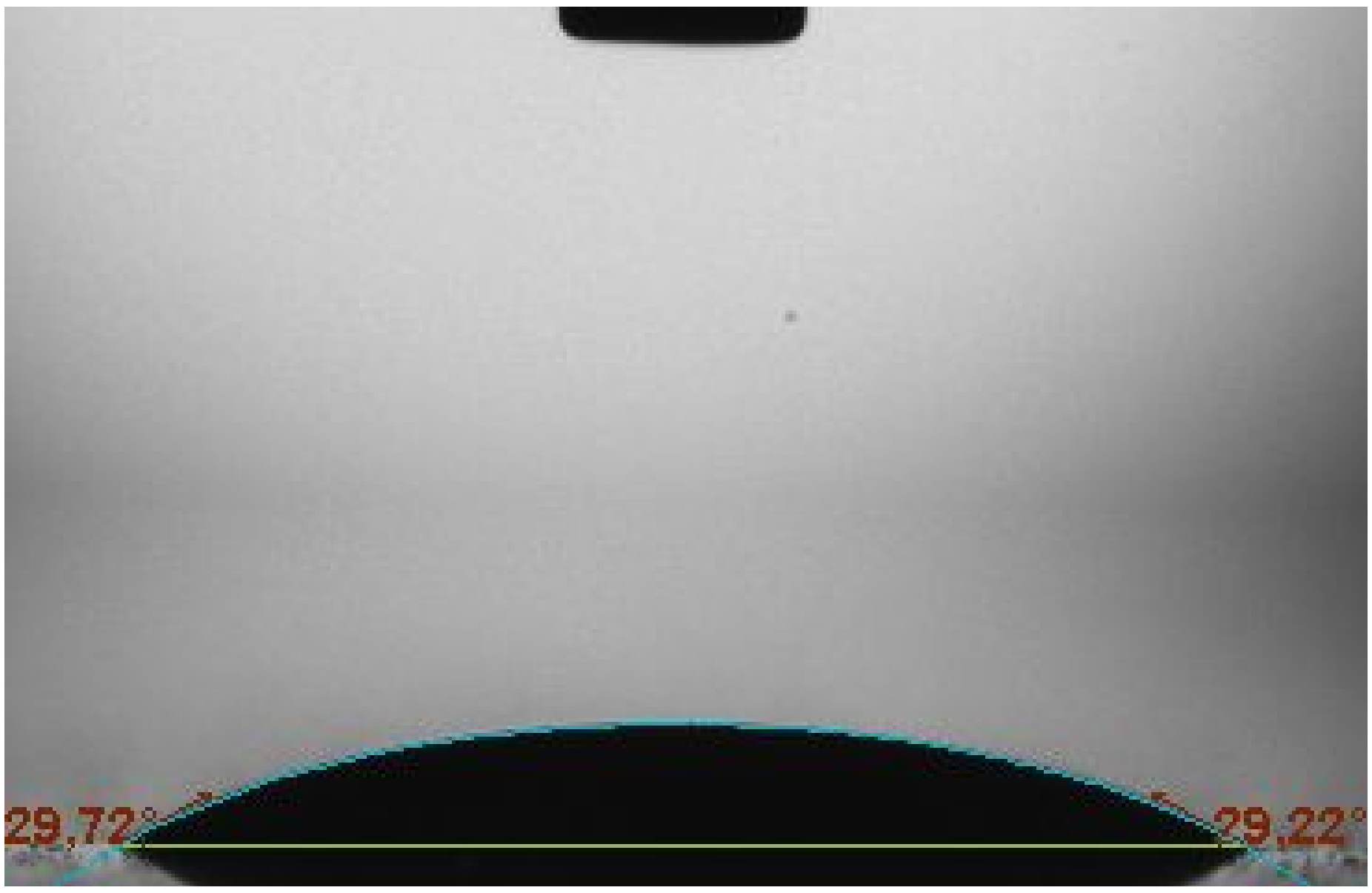

Figure S3. Contact Angle Measurement of Graphene Ink on PET Substrate. The measured contact angle of 29° confirms the high wettability of the graphene ink on the PET substrate, facilitating high-quality printing.

Table S1. Comparative analysis of the proposed inkjet-printed graphene electrodes relative to state-of-the-art graphene-based biopotential sensors.

**Ref.**	**Method**	**Substrate**	**Biopotential**	**Patterning Flexibility**	**Sustainability**
**This Work**	Inkjet	PET	ECG, EEG, EMG, EOG	Digital / High	Recyclable, Waste-free, multi-use
Yapici et al. (2015)[1]	Dip-coat	Textile	ECG	None	Multi-use
Karim et al. (2017) [2]	Inkjet	Textile	ECG	Digital / High	Waste-free, multi-use
Liang et al. (2022) [3]	Dyeing	Textile	ECG, EMG	None	Recyclable, multi-use
Kwon et al. (2020) [4]	Aerosol Jet Printing	PI	EMG	High / Digital	Waste-free, multi-use
Kireev et al. (2021) [5]	CVD	PMMA	ECG, EEG, EMG, EOG	None	Recyclable
Zahed et al. (2020) [6]	Laser-induced	PI	ECG	Digital / High	Waste-free, multi-use
Xu et al. (2020) [7]	Screen-print	Textile	ECG	Physical mask / Low	multi-use

Table S2. Demographic characteristics of ten participants for ECG signal recordings.

Participant	Gender	Height	Weight	Age	BMI
1	Male	191 cm	83 Kg	30	22.8
2	Female	165 cm	59 Kg	27	21.7
3	Male	179 cm	73 Kg	31	22.8
4	Male	178 cm	60 Kg	34	18.9
5	Female	165 cm	67 Kg	25	24.6
6	Male	187 cm	94 Kg	36	26.9
7	Female	177 cm	60 kg	22	19.15
8	Female	172 cm	78 kg	26	26.4
9	Male	179 cm	85 kg	30	26.5
10	Female	169 cm	82 kg	25	28.7

Movie S1. Graphene Inkjet Printing: Jetting Profile. This movie highlights the highly controllable and precise drop-on-demand graphene jetting profile of 4 out of 12 nozzles.

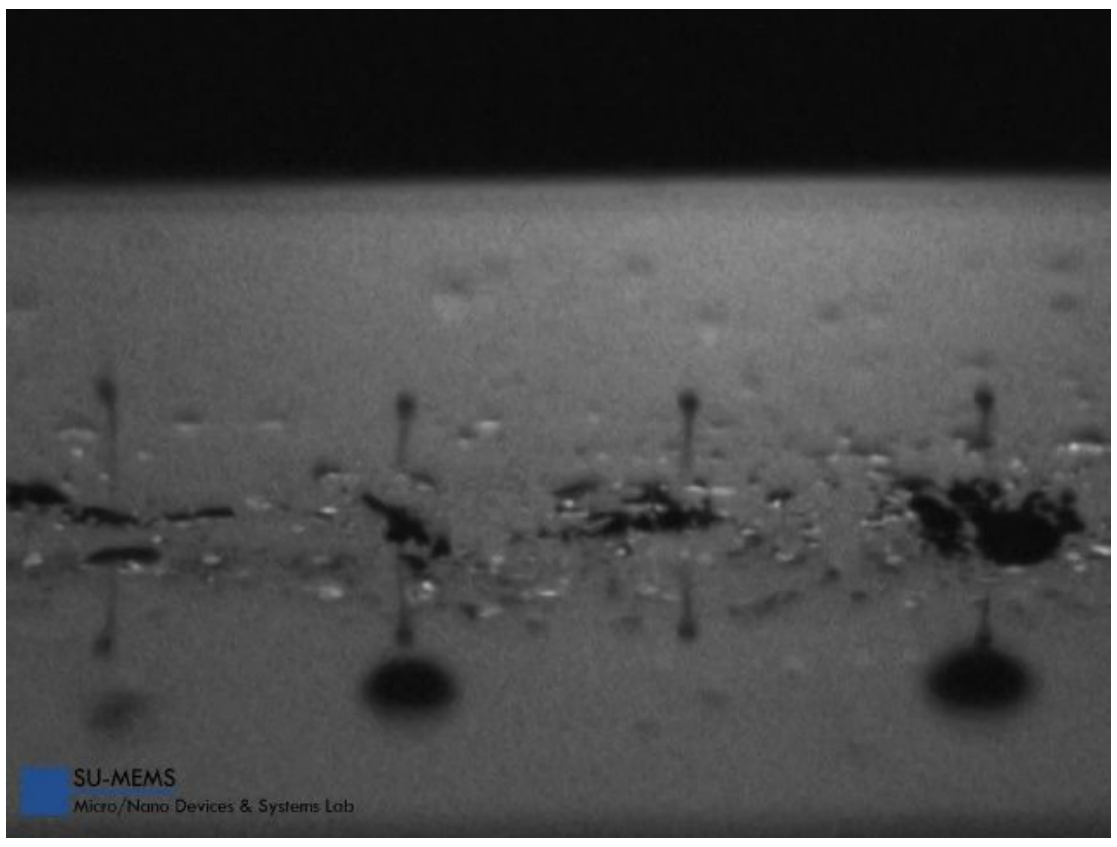

Movie S2. Resistance Stability of Printed Graphene Electrodes Under Cyclic Bending. This movie demonstrates the flexibility of printed graphene electrodes by measuring resistance variations during mechanical deformations, under 100 cycles of bending.

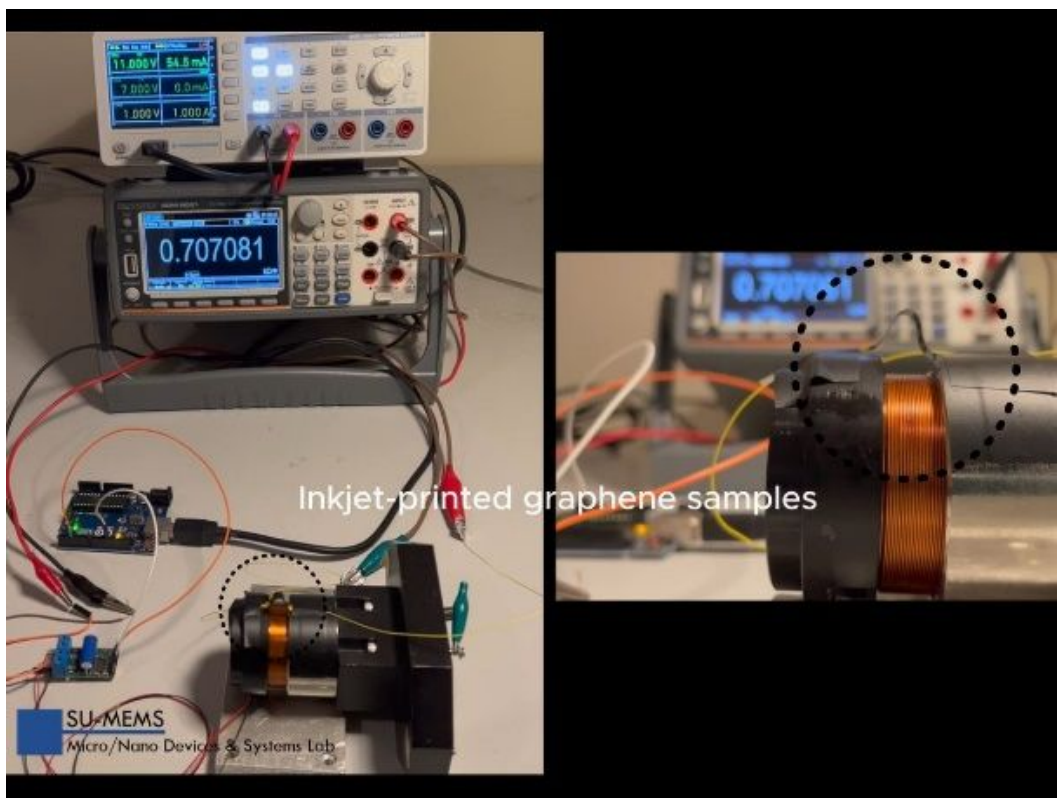

Movie S3. Adhesion Analysis of Inkjet-Printed Graphene on PET Using Scotch Tape Experiment and Resistance Measurement. This movie demonstrates the adhesion quality of printed graphene ink on PET substrates through a qualitative Scotch-tape peel test, repeated four times, while measuring the sample's resistance throughout the process.

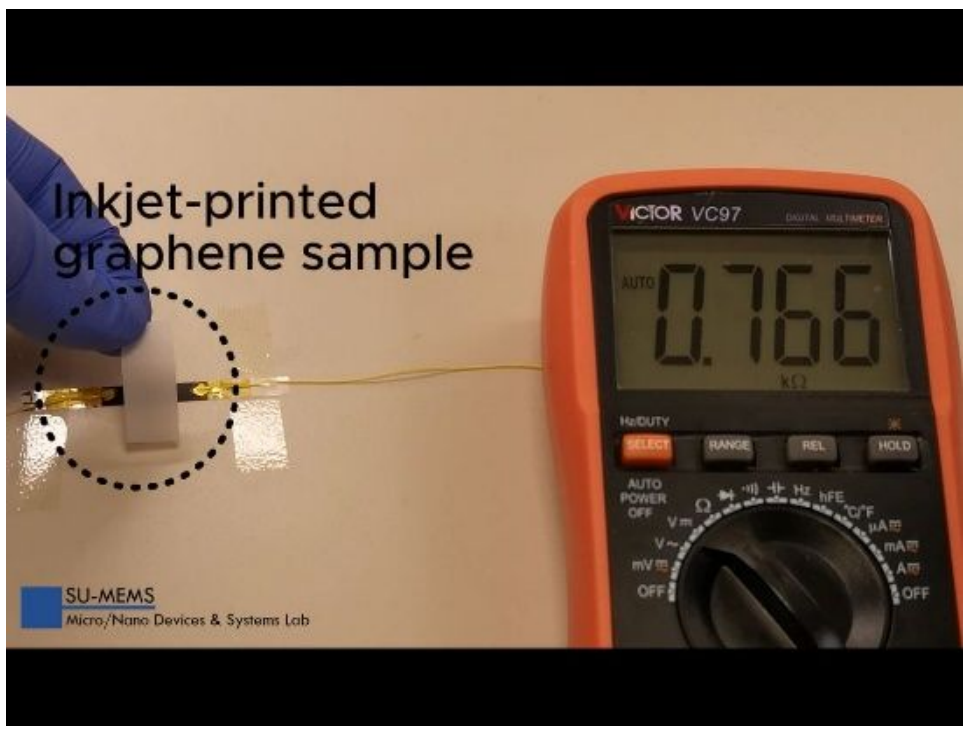

Movie S4. EEG Safety System to Detect and Alert Drowsy Drivers. This movie shows the detection of Alpha waves in EEG signals generated a few seconds after transitioning from open to closed eyes using graphene electrodes. The proposed system provides auditory stimuli to alert drivers in cases of drowsiness, waking them to prevent fatal accidents.

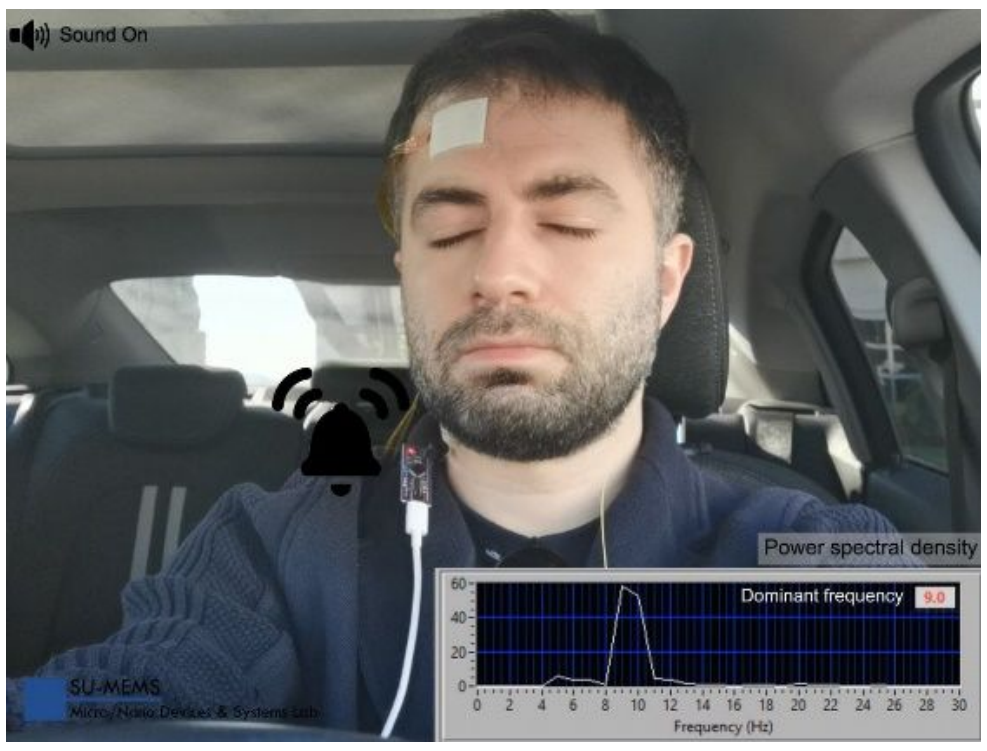

References

(1) Yapici, M. K.; Alkhidir, T.; Samad, Y. A.; Liao, K. Graphene-Clad Textile Electrodes for Electrocardiogram Monitoring. *Sens. Actuators B Chem.* **2015**, *221*, 1469–1474. https://doi.org/10.1016/j.snb.2015.07.111.

(2) Karim, N.; Afroj, S.; Malandraki, A.; Butterworth, S.; Beach, C.; Rigout, M.; Novoselov, K. S.; Casson, A. J.; Yeates, S. G. All Inkjet-Printed Graphene-Based Conductive Patterns for Wearable e-Textile Applications. *J. Mater. Chem. C Mater.* **2017**, *5* (44), 11640–11648. https://doi.org/10.1039/C7TC03669H.

(3) Liang, X.; Zhu, M.; Li, H.; Dou, J.; Jian, M.; Xia, K.; Li, S.; Zhang, Y. Hydrophilic, Breathable, and Washable Graphene Decorated Textile Assisted by Silk Sericin for Integrated Multimodal Smart Wearables. *Adv. Funct. Mater.* **2022**, *32* (42). https://doi.org/10.1002/adfm.202200162.

(4) Kwon, Y.-T.; Kim, Y.-S.; Kwon, S.; Mahmood, M.; Lim, H.-R.; Park, S.-W.; Kang, S.-O.; Choi, J. J.; Herbert, R.; Jang, Y. C.; Choa, Y.-H.; Yeo, W.-H. All-Printed Nanomembrane Wireless Bioelectronics Using a Biocompatible Solderable Graphene for Multimodal Human-Machine Interfaces. *Nat. Commun.* **2020**, *11* (1), 3450. https://doi.org/10.1038/s41467-020-17288-0.

(5) Kireev, D.; Ameri, S. K.; Nederveld, A.; Kampfe, J.; Jang, H.; Lu, N.; Akinwande, D. Fabrication, Characterization and Applications of Graphene Electronic Tattoos. *Nat. Protoc.* **2021**, *16* (5), 2395–2417. https://doi.org/10.1038/s41596-020-00489-8.

(6) Zahed, M. A.; Das, P. S.; Maharjan, P.; Barman, S. C.; Sharifuzzaman, M.; Yoon, S. H.; Park, J. Y. Flexible and Robust Dry Electrodes Based on Electroconductive Polymer Spray-Coated 3D Porous Graphene for Long-Term Electrocardiogram Signal Monitoring System. *Carbon N. Y.* **2020**, *165*, 26–36. https://doi.org/10.1016/j.carbon.2020.04.031.

(7) Xu, X.; Luo, M.; He, P.; Yang, J. Washable and Flexible Screen Printed Graphene Electrode on Textiles for Wearable Healthcare Monitoring. *J. Phys. D Appl. Phys.* **2020**, *53* (12), 125402. https://doi.org/10.1088/1361-6463/ab5f4a.
